# Interdisciplinary Management of White Coat Hypertension in Geriatric Oral Surgery: Case Report

**DOI:** 10.3390/geriatrics10060172

**Published:** 2025-12-18

**Authors:** Alexandra Allaica Cuenca, Ana Balseca Morales, Jorge López Bundschuh, Luis Chauca-Bajaña, Byron Velasquez Ron

**Affiliations:** 1 Carrera de Odontología, Facultad de Odontología, Universidad de Las Américas (UDLA), Colon y 6 de Diciembre, Quito 170516, Ecuador; nicole.allaica.17heac@gmail.com (A.A.C.); ana.balseca@udla.edu.ec (A.B.M.); jorge.lopez@udla.edu.ec (J.L.B.); 2 College Dentistry, Universidad de Guayaquil, Guayaquil 090101, Ecuador; 3 Department Prosthesis Research, Universidad de Las Américas (UDLA), Av. Colón y 6. Diciembre, Quito CP 170516, Ecuador

**Keywords:** white coat hypertension, blood pressure, conscious sedation, psychological intervention, oral surgery

## Abstract

Introduction: White coat hypertension in geriatric patients can complicate dental procedures in the presence of intense anxiety. Objective: To evaluate the effectiveness of a combined approach of psychological intervention and sedation for the control of the syndrome during multiple extractions. Case presentation: A 76-year-old woman with a diagnosis of white coat hypertension (WCH) and a history of dental anxiety. In two previous attempts, the surgery was suspended due to blood pressure elevation. The Dental Perception Reprogramming Protocol (DPRP) was applied along with conscious sedation (midazolam, fentanyl, dexmedetomidine) which allowed agitation, so deep sedation with propofol was used. Result: The patient had stable blood pressure (119/82 mmHg) and successfully completed the intervention without complications. Conclusions: The integration of psycho-behavioral and pharmacological techniques allowed effective hemodynamic control, and a key interdisciplinary approach is suggested for the management of the syndrome in older adults.

## 1. Introduction

In dentistry, a negative experience in childhood or youth can intensify stress and anxiety levels [1], which can lead to healthcare phobia. This response manifests itself in three systems: motor, cognitive and physiological. The latter is evident with a transient increase in blood pressure, caused by the activation of the sympathetic system, which raises cardiac output at the systolic level, generating a hypertensive response [2]. This phenomenon is known as white coat hypertension (WCH), reactive hypertension or isolated clinical hypertension, and is characterized by an increase in blood pressure in the clinic greater than 140/90 mmHg, with normal values of less than 135/85 mmHg in ambulatory monitoring in patients without hypertensive medication [3]. Reactive hypertension tends to worsen in geriatric patients due to the decrease in vascular elasticity typical of aging [4,5], increasing the probability of developing hypertension and cerebrovascular diseases [6,7]. Based on this approach, the hypothesis is proposed that the combination of psychological intervention and conscious sedation can effectively control WCH during dental surgical procedures. The objective was to evaluate the effectiveness of a combined approach of psychological intervention and sedation for the control of white coat hypertension (WCH) during multiple extractions.

## 2. Case Report

This case report was prepared in accordance with the CARE guidelines. A 76-year-old female patient went to the Dental Care Center of the University of the Americas (CAO-UDLA) due to a fracture of a fixed prosthesis (dental bridge). There is a personal history of hypothyroidism secondary to the removal of a benign thyroid nodule approximately eight years ago, which is currently controlled with levothyroxine 50 mcg/day in the mornings. She also has hypercholesterolemia, treated with simvastatin 20 mg/day at night.

**Figure 1:**
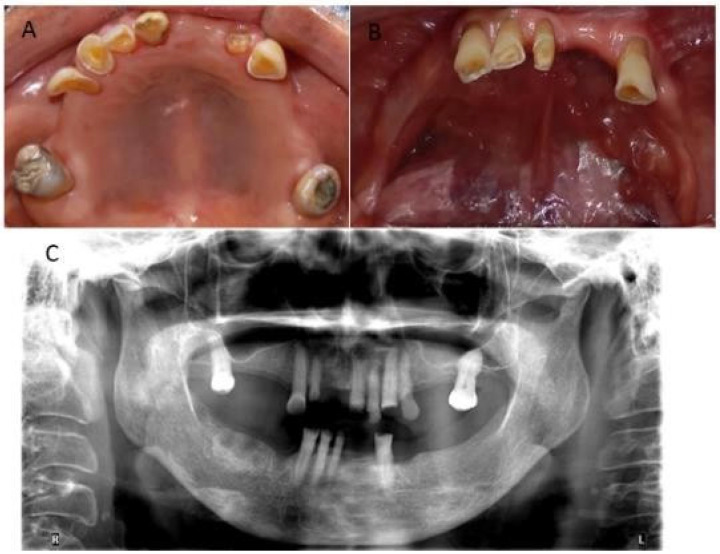
Upper (**A**) and lower (**B**) occlusal intraoral photography and (**C**) panoramic radiograph.

Extraoral examination identified a dolichofacial biotype with a convex profile, the presence of deep nasolabial folds, lip hypotonicity, and a noticeable reduction of the lower third of the face, which clinically suggests a loss of vertical dimension. This impression was confirmed in the intraoral examination, where the absence of multiple teeth and, as a consequence, a loss of subsequent containment was evidenced. The remaining pieces show wear and functional alterations, such as abfraction, attrition, mobility, and generalized periodontal involvement (Figure 1A,B).

In both jaws, the crown–root ratio was compromised, with a negative prognosis of teeth, indicating that multiple teeth required extraction and regularization of alveolar ridges. Prosthetic rehabilitation after surgery and immediate conventional removable total prostheses allow us to avoid the edentulous period and favor the patient’s self-esteem, facilitating their adaptation, providing protection to the post-extraction alveoli, and acting as a surgical splint during healing. The procedure began with taking impressions using alginate (TROPICALGIN–Zhermack, Badia Polesine, Rovigo, Italia) in perforated metal trays (size L) for the maxilla and mandible, respectively. Casting was performed with type III stone plaster (Whipmix, Colone, Germany) to obtain the working models, and base wax buns were made for the intermaxillary recording.

**Figure 2:**
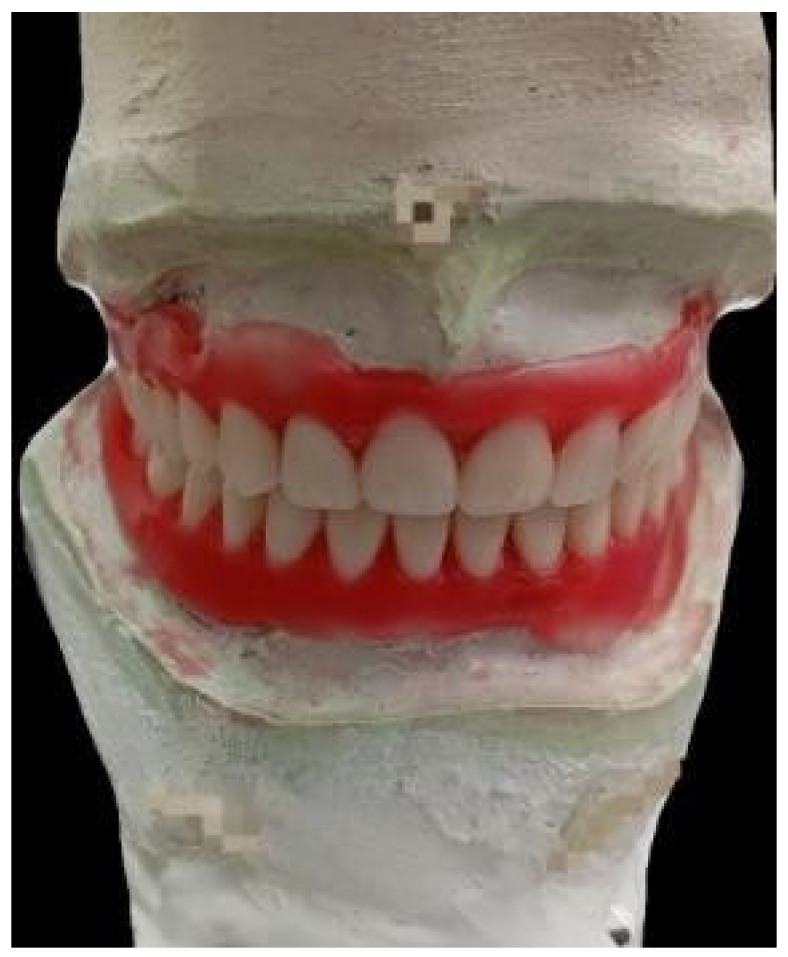
Fixation on the articulator with bilateral balanced occlusion, after model surgery and prior to acrylization.

We recorded a Vertical Dimension at Rest (DVR) of 6.8 cm and a Vertical Dimension at Occlusion (DVO) of 6.3 cm; it was decided to increase these by 2 mm, with an interocclusal clearance of 3 mm, using maxillomandibular recording in conjunction with Dawson’s bimanual technique. Articulated models of the facial arch were created in an A7 Plus semi-adjustable articulator (Bio-Art, São Carlos, São Paulo, Brasil) for the transfer of records. The models were created with the aim of carrying out a clinical test to evaluate occlusal stability. Functionality and stability were confirmed, and we proceeded to perform model surgery (Figure 2) after the acrylization process.

Pre-surgical laboratory tests, including blood count, blood chemistry and coagulation tests, were performed in order to prevent bleeding and evaluate healing capacity(Table 1).

During the second surgical attempt, the patient presented stage 2 hypertension (159/84 mmHg) during the extraction of teeth 41 and 42, which decreased to 146/82 mmHg after stopping the procedure. Due to this unstable response, the session was suspended and the patient was referred to internal medicine and cardiology for evaluation. After specialist assessment, a diagnosis of reactive hypertension or white coat hypertension (WCH) was confirmed, as elevated values occurred exclusively during dental treatment.

Blood pressure was measured using an automated oscillometric digital monitor (OMRON HEM-7120, VIETNAM Co., Ltd., Thu Dau Mot City, Vietnam, clinically validated) with the patient seated, feet supported, and the arm positioned at heart level. In each session, baseline measurements were taken and subsequently repeated every 5 min during the pre-surgical phase. During sedation and surgery, non-invasive BP cycling was set to 3–5 min intervals. Whenever an elevated value was detected, two additional measurements were obtained 1 min apart, and the lowest of the three was recorded, following international hypertension monitoring recommendations. All measurements were performed by a trained operator.

A third intervention was planned, this time integrating psychological support and a structured behavioral protocol to prevent recurrent hypertensive episodes. At the beginning of this session, blood pressure was 127/81 mmHg; however, during the extraction of tooth 43, it rose abruptly to 186/83 mmHg, corresponding to stage 3 hypertension (hypertensive crisis), although without clinical symptoms. Considering the two previous hypertensive events, it was decided to incorporate the Dental Perception Reprogramming Protocol (DPRP) together with conscious sedation to reduce anxiety-driven sympathetic activation and improve hemodynamic stability during the surgical procedure (Table 2).

For this session, the combined use of 2% mepivacaine with 1:100,000 epinephrine and 3% mepivacaine without epinephrine was planned in order to reduce the total load of vasoconstrictor administered. This decision was based on the immunosuppressed conditions of the patient, controlled hypothyroidism, and white coat hypertension without evidence of heart damage. The partial use of anesthesia without vasoconstrictor ensures adequate intraoperative pain control, while optimizing cardiovascular safety during the procedure.

**Figure 3:**
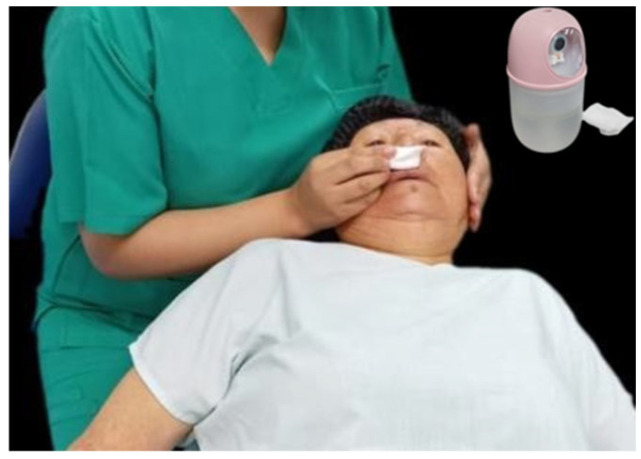
Aromatherapy with lavender applied with humidifier and gauze.

At the beginning of the preoperative phase, the patient presented a blood pressure of 195/95 mmHg, corresponding to stage 3 arterial hypertension, without symptoms of hypertensive crisis. The psycho-behavioral protocol was initiated by beginning with aromatherapy, placing lavender essential oil on gauze for the patient to inhale, while guided by a directed visualization. The phrases used in the conversation were “You are entering a place where there is no pain, only curiosity. Here everything flows slowly and you are safe.” (Figure 3). Lavender acts on olfactory receptors directly connected to the limbic system, responsible for regulating emotions and memory; its anxiolytic effect is widely demonstrated. Guided imagery helps interrupt the cycle of anticipatory anxiety.

The protocol continued with a soothing facial stimulation technique (tapping), gentle tapping with the index and middle fingers on the temples and cheekbones for approximately 10 s, while the patient spoke. This gesture should appear natural and empathetic, not an obvious therapeutic technique. Tapping activates reflex relaxation pathways, calming the response of the nervous system in the patient. Blood pressure was reduced to 172/80 mmHg; this decrease was insufficient. This was probably because in states of emotional hyperalertness such as intense anxiety and negative emotional memory related to the dental environment, a sustained sympathetic activation is generated that is difficult to control with behavioral techniques alone. This situation is more common in older adult patients, such as in this case, and in those who have a more rigid vascular tone and a lower response to rapid relaxation mechanisms.

**Figure 4:**
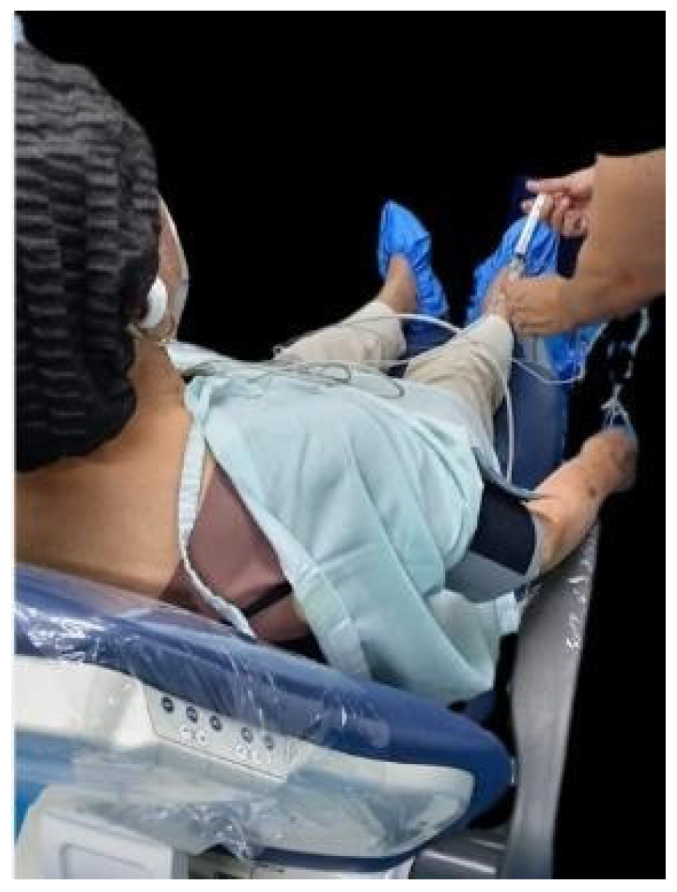
Administration of IV fentanyl.

Given the ineffectiveness of the psycho-behavioral interventions applied, it was decided to administer fentanyl 50 mcg intravenously, adjusted for the patient’s body weight (Figure 4). This opioid, in addition to its analgesic effect, acts at the level of the central nervous system by attenuating sympathetic activity, which reduces the release of endogenous catecholamines. This mechanism, together with its mild peripheral vasodilatory action, favors a decrease in vascular resistance and general tone, allowing better hemodynamic control; blood pressure dropped to 119/82 mmHg, allowing the intervention to continue in a safe and controlled manner.

During the surgical phase, the protocol was continued with audio analgesia using binaural music at Theta frequencies (4–8 Hz), played with headphones. These frequencies are associated with states of deep relaxation, reduce anxiety, and favor the effect of conscious sedation by synchronizing brain activity. As a complementary stimulus, aromatherapy with lavender was maintained using a humidifier. This aroma, previously used in the pre-surgical phase, acts as an olfactory anchor of relaxation to associate the beginning of the protocol with a state of calm and emotional security. Its repetition during the surgical phase reinforces this connection, consolidating a secure emotional imprint that contributes to maintaining the patient’s emotional and physiological stability during the procedure. Pharmacological management of anxiety was intravenously administered with dexmedetomidine and midazolam, which act through different but complementary mechanisms. Midazolam, a short-acting benzodiazepine, boosts the activity of the neurotransmitter GABA in the central nervous system, generating anxiolytic, sedative and amnesic effects. Dexmedetomidine is a selective agonist of alpha-2 adrenergic receptors that acts primarily on the locus coeruleus, inhibiting the release of norepinephrine and reducing sympathetic activity. This combination produces cooperative sedation, with anxiolysis and analgesia, without compromising respiratory function. The patient reached a state of moderate conscious sedation, with a score of -3 on the Richmond Agitation–Sedation Scale (RASS), presenting movements and eye opening to verbal call, but without eye contact. In addition, ketorolac and paracetamol (30 mg IV each) were applied to control pain, and ondansetron (4 mg IV) and dexamethasone (8 mg IV) were used to prevent nausea and vomiting, reducing postoperative inflammation. After the placement of infiltrative anesthesia in the area of the teeth removed, the patient presented an episode of intense agitation, trying to remove the catheter and the surgical drape, with a score of +3 on the RASS (severe agitation). Given this reaction, and to ensure the safety of the patient during the procedure, the team decided to administer propofol by IV; it has a stronger and faster effect. It acts by directly enhancing the action of GABA in the central nervous system, prolonging the opening of chlorine channels and generating a more extensive neuronal depression. Its administration induced a state of deep sedation, in which the patient did not respond to verbal call and only reacted to painful stimuli, corresponding to a score of −4 on the RASS (Table 3).

**Figure 5:**
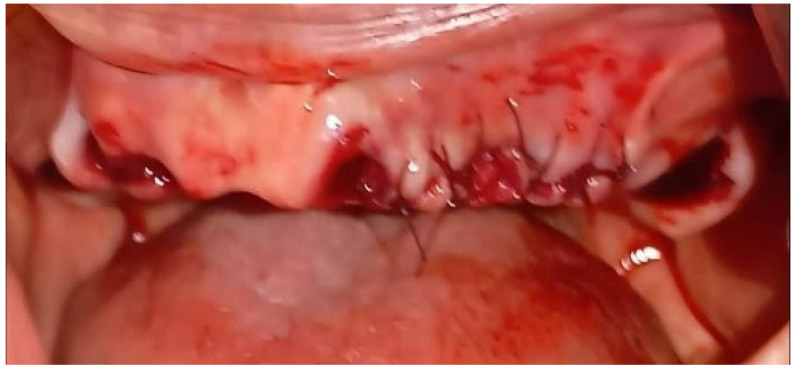
Sutured anterior superior area.

Continuing with the intervention, three anesthetic cartridges were administered: two with a vasoconstrictor (2% mepivacaine with 1:100,000 epinephrine) and one without a vasoconstrictor (3% mepivacaine), respecting physiological limits. All nine teeth were removed without complications. In the corresponding alveoli of teeth 17, 27 and 33, hemostatic sponges were placed to promote local coagulation. Bone regularization was performed in the upper jaw due to the presence of bone spicules. Finally the area was sutured with Vicryl 3-0 resorbable thread (Figure 5).

**Figure 6:**
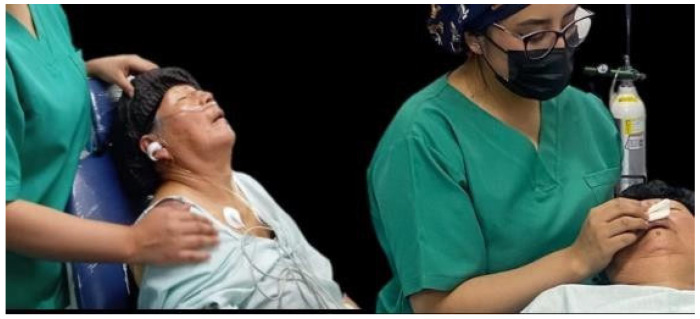
Emotional narrative and aromatherapy.

In the post-surgical phase, a directed emotional narrative was used, speaking in a low and calm voice during recovery: “You did very well, your body remembers this as a safe and calm experience, very different from other times.” With the effects of light sedation still, the brain continues to register verbal information, which allows the emotional interpretation of the event to be reprogrammed, reducing the risk of anxiety or phobia in the future. It is sensorily reinforced, applying the scent of lavender to act as a familiar and calming stimulus that helps to consolidate a positive perception of the dental environment (Figure 6).

Complementing the intravenous pharmacological management administered during surgery, granulated paracetamol 500 mg every 8 h for 5 days was prescribed as an analgesic and amoxicillin 500 mg every 8 h for 7 days as an oral antibiotic. In this third intervention, the surgical phase of the treatment plan was successfully completed, highlighting better control of blood pressure compared to previous sessions (Table 4).

To provide a clearer quantitative overview of the patient’s hemodynamic response across the three interventions, simple descriptive statistics and percentage changes were calculated using the values presented in Table 4. During the first intervention, the surgical systolic BP reached 159 mmHg. In the second intervention, despite guided breathing, systolic BP increased to 186 mmHg, representing a 17% rise compared to the first attempt. In contrast, during the third intervention, surgical systolic BP decreased to 119 mmHg—approximately 25% lower than in the first intervention and 36% lower than in the second.

Pre-surgical systolic BP also showed progressive improvement. It decreased from 195 mmHg to 172 mmHg with the application of the DPRP alone (a 12% reduction), and further to 119 mmHg following pharmacological management (a total reduction of 39%).

These descriptive values highlight a consistent downward trend in sympathetic activity and improved hemodynamic stability across the three sessions. While inferential statistical analysis is not applicable in a single-patient design, these changes demonstrate clinically meaningful effect sizes. Future research may incorporate n-of-1 trial methodologies to quantitatively evaluate individualized treatment effects.

**Figure 7:**
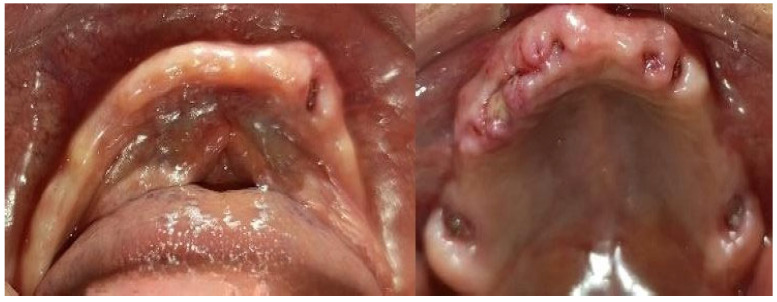
Healing after 15 days.

Continuing with the prosthetic planning, the immediate prostheses were placed the day after the surgical procedure, using Ufigel (Voco/Cologne/Germany) as temporary support material to improve comfort and initial adaptation, acting as a soft interface on the post-surgical tissues. The stitches were removed after 15 days, and good healing of the operated areas and an adequate adaptation of the prostheses were observed without complications (Figure 7).

## 3. Discussion

White coat hypertension (WCH) can be understood as a psychosomatic manifestation of clinical anxiety in medical contexts [8]. In dentistry, this phenomenon tends to intensify, especially in geriatric patients who have had previous negative experiences, increasing cardiovascular risk during surgical procedures. In this case, the application of a psycho-behavioral protocol, the Dental Perception Reprogramming Protocol (DPRP), is presented for the first time, designed to modulate the physiological response to the surgical environment in patients with WCH [9]. This protocol integrates multisensory techniques such as aromatherapy, audio analgesia, cognitive strategies such as guided imagery, directed storytelling, and behavioral methods such as tapping. All of these tools are applied sequentially during the pre-surgical, surgical, and post-surgical phases.

In aromatherapy, the use of lavender helps to significantly reduce anxiety, blood pressure, and heart rate in patients during dental treatments. Recent clinical studies have confirmed these effects with a high level of statistical certainty [10]. Theta frequency-based audio analgesia (4–8 Hz) has been shown to be effective in inducing a state of deep relaxation, contributing to better emotional regulation in clinical settings. Research such as that of Janthasila/Keeratisiroj [11] shows that the combination of therapeutic music and aromatherapy not only decreases anxiety and blood pressure, but also the perception of pain during dental procedures.

The facial tapping technique was incorporated, a form of gentle somatosensory stimulation that has shown benefits in interrupting the state of sympathetic hyperalertness. Its mechanism of action is still under study; it is believed that it can activate reflex parasympathetic responses by regulating the hypothalamic–pituitary–adrenal axis [9]. One of the most important pillars of DPRP is the postoperative emotional narrative, which is based on principles of emotional memory reconsolidation, which indicates that the brain can reinterpret events even under light sedation, forming new emotional associations without the need for full patient awareness [12]. Applied to the clinical setting, this approach allows for a resignification of the surgical experience, reducing future emotional rejection and strengthening experiences in treatments to be performed.

Conscious sedation used midazolam, fentanyl and dexmedetomidine, a combination that produces anxiolysis, analgesia and cooperation [13]. In this case, reflex movements and agitation persisted during anesthetic infiltration. The relative ineffectiveness of conscious sedation can be attributed to the intensity of the emotional component (negative somatic memory) [14] and a resistant sympathetic response, which is common in geriatric patients with severe dental anxiety [15]. Recent literature recognizes that midazolam is useful for moderate sedation, but has limited effects on sympathetic axis suppression in high-stress scenarios [16]. The lack of response invited the use of deep sedation with propofol, which has powerful GABAergic effects, the ability to completely suppress motor reactivity, and a safe profile when administered in controlled boluses [17]. The sequence of interventions allowed the procedure to be developed with hemodynamic stability, thanks to the gradual integration of psychological and pharmacological resources adjusted to the case.

Confounding Factors and Comorbidity Considerations

The interpretation of hemodynamic outcomes in this case must take into account several potential confounders. The patient’s comorbidities—particularly hypothyroidism, age-related vascular rigidity, and chronic hypercholesterolemia—may influence baseline autonomic regulation and the physiological response to sedative medications. Older adults commonly show increased sensitivity to benzodiazepines and dexmedetomidine, as well as slower drug clearance, which may potentiate sedative depth and hemodynamic fluctuations. In addition, severe dental anxiety and negative emotional memory acted as psychological confounders that likely amplified sympathetic activation despite pharmacological sedation. Because the DPRP and sedatives were applied sequentially, their individual contributions cannot be fully isolated, and synergistic effects between behavioral and pharmacological interventions remain possible. Future controlled studies should account for these variables to better understand interaction effects between comorbidities, anxiety severity, and sedation regimens.

Limitations and Recommendations for Future Research

This case report presents the successful interdisciplinary management of white coat hypertension in a single geriatric patient undergoing oral surgery. However, several limitations must be acknowledged. First, the findings cannot be generalized, as individual psychological responses, hemodynamic variability, and sensitivity to sedative drugs may differ significantly among older adults. Second, the physiological effect of the Dental Perception Reprogramming Protocol (DPRP) cannot be isolated from the pharmacological intervention, since both were used sequentially. Therefore, the degree to which each component contributed to blood pressure stabilization cannot be independently quantified. Finally, this study lacks long-term follow-up to determine whether the emotional reconditioning achieved through DPRP persists over time and reduces blood pressure reactivity in future dental procedures.

Future research should include larger case series or controlled clinical studies to evaluate the reproducibility of the DPRP in different populations and settings. Comparative studies between psychological-only, pharmacological-only, and combined approaches would help establish the relative contribution of each component. Additionally, physiological monitoring tools such as heart rate variability analysis could provide objective biomarker data to better understand autonomic modulation in white coat hypertension during dental care.

Rationale and Development of the Dental Perception Reprogramming Protocol (DPRP)

The Dental Perception Reprogramming Protocol (DPRP) was conceptualized as a structured, multisensory behavioral intervention designed to modulate anticipatory anxiety and autonomic hyperreactivity in patients with WCH. The protocol integrates components derived from cognitive–behavioral therapy (CBT), guided imagery, clinical communication strategies, and principles of emotional memory reconsolidation. The therapeutic phrases used—such as “You are entering a place where there is no pain, only curiosity”—are adapted from CBT-based cognitive reframing techniques commonly employed to shift maladaptive threat-based perceptions toward neutral or exploratory interpretations. The sequence of sensory inputs (aromatherapy → guided imagery → tapping → auditory entrainment → narrative reframing) was organized to progressively downregulate sympathetic activity, beginning with low-cognitive-load stimuli and advancing toward higher-order cognitive restructuring once the patient reached a receptive state.

Although DPRP is not yet a validated psychological protocol, its components are evidence-based individually, and the structured combination was developed empirically through interdisciplinary collaboration between dentistry, behavioral science, and anesthesia. The protocol currently relies on a semi-standardized script, with each phase lasting approximately 3–5 min, and requires providers to be trained in guided communication and anxiety-modulation techniques. Future studies are needed to standardize timing, optimize scripting, and evaluate physiological outcomes to support broader clinical implementation.

Confounding Factors and Comorbidity Interactions

The interpretation of the hemodynamic changes in this case must consider several potential confounding factors. The patient’s comorbidities—particularly age-related vascular rigidity, hypothyroidism, hypercholesterolemia, and severe dental anxiety—can influence autonomic reactivity and modify the pharmacodynamic response to sedative medications. Older adults often exhibit increased sensitivity and slower clearance of midazolam, dexmedetomidine, fentanyl, and propofol, which may potentiate their hemodynamic effects. Furthermore, because the behavioral intervention (DPRP) and pharmacological sedation were applied sequentially, their individual contributions cannot be fully isolated, and synergistic interactions may have occurred. These factors limit the ability to attribute the observed blood pressure stabilization to a single component of the intervention.

## 4. Conclusions

The initial implementation of the Dental Perception Reprogramming Protocol (DPRP) allowed us to partially reduce anxiety, but it did not manage to stabilize blood pressure on its own. Subsequent administration of conscious sedation with midazolam, fentanyl, and dexmedetomidine facilitated surgical cooperation, although reflex motor responses persisted. Deep sedation under propofol made it possible to complete the procedure without hypertensive events. This progression of management confirms that in patients with WCH, tension regulation requires a comprehensive and personalized approach due to the link between emotions and physiological responses. Combined strategies can offer a humanized clinical surgical path.

## 5. Clinical Implications

This case highlights the importance of integrating psychological strategies, such as Dental Perception Reprogramming Protocol (DPRP), with staged sedation in geriatric patients with white coat hypertension. This approach can reduce anxiety, improve patient cooperation, and maintain hemodynamic stability during complex surgical procedures. The findings suggest that interdisciplinary protocols combining emotional and pharmacological management may enhance patient safety, efficiency, and overall experience in oral surgery.

## Figures and Tables

**Table 1:** Coagulation test results.

Test	Results	References Values
TTP	25.10 seg	20.00–35.00 seg
TP	11.40 seg	11.00–14.50 seg
INR	0.94	-
Coagulation Time	13 min	5.00–10.00 min

**Table 2:** Dental Perception Rescheduling Protocol.

Phase	Main Technique	Physiological/Emotional Goal
Pre-surgical	Aromatherapy and Guided Imagery + Tapping	Decrease sympathetic tone, break negative anticipation
Surgical	Audioanalgesia + matching aroma	Multisensory stimulation of Subconscious Security
Post-surgical	Narrative + aromatherapy	Post Positive Emotional Coding procedure

**Table 3:** Summary of the sedation protocol applied during the third intervention.

Medication	Dose	Purpose/Mechanism	Clinical Role in This Case
Fentanyl (IV)	50 mcg	Opioid analgesic; decreases sympathetic tone	Initial control of anxiety-related BP elevation
Midazolam (IV)	Titrated	Benzodiazepine; anxiolysis, amnesia, sedation	Achieved moderate conscious sedation
Dexmedetomidine (IV)	Infusion	α2-agonist; reduces norepinephrine release	Stabilizes hemodynamics
Propofol (IV)	Bolus	GABAergic hypnotic	Used after severe agitation (RASS + 3)
Ketorolac (IV)	30 mg	Analgesic (NSAID)	Intraoperative pain control
Paracetamol (IV)	30 mg	Analgesic	Supplemental analgesia
Ondansetron (IV)	4 mg	Antiemetic	Prevents nausea/vomiting
Dexamethasone (IV)	8 mg	Corticosteroid	Reduces postoperative inflammation

**Table 4:** Patient evolution in the three interventions.

Parameter	First Intervention	Second Intervention	Third Intervention
Knowledge of WCH	no	yes	yes
Applied technique	Controlled environment	Guided breathing	DPRP Sedación consciente
Pre-surgical BP	138/80 mmHg	135/79 mmHg	195/95 mmHg
Pre-surgical BP without pharmacological management	-	127/81 mmHg	172/80 mmHg
Pre-surgical BP with pharmacological management	-	-	119/82 mmHg
Surgical BP	159/84 mmHg	186/83 mmHg	119/82 mmHg
Post-surgical BP	146/82 mmHg	143/83 mmHg	123/81 mmHg
Tooths removed	2	1	9
Procedure completed	Partial	Partial	Total
Result	Hypertension not detected, partial intervention	WCH identified, BP not controlled, intervention limited	WCH controlled. Procedure completed without complications.

## Data Availability

The original contributions presented in this study are included in the article. Further inquiries can be directed to the corresponding author.

## Abbreviation

Abbreviation	Meaning
WCH	White Coat Hypertension
DPRP	Dental Perception Reprogramming Protocol
mmHg	Millimeters of Mercury
CAO-UDLA	Dental Care Center, Universidad de Las Américas
mcg	Microgram
mg	Milligram
DVR	Vertical Dimension at Rest
DVO	Vertical Dimension in Occlusion
cm	Centimeter
mm	Millimeter
PTT	Partial Thromboplastin Time
PT	Prothrombin Time
INR	International Normalized Ratio
seg	Second
min	Minute
IV	Intravenous
Hz	Hertz
GABA	Gamma-Aminobutyric Acid
RASS	Richmond Agitation-Sedation Scale
BP	Blood Pressure
AHT	Arterial Hypertension
